# Veterinary Medical Association of New York County

**Published:** 1896-03

**Authors:** R. W. Ellis

**Affiliations:** Secretary


Secretary.

### VETERINARY MEDICAL ASSOCIATION OF NEW YORK COUNTY.

The regular meeting was held on Tuesday evening, February 4th, at 8.30 o'clock, with the President, Dr. Huidekoper, in the chair. On roll-call the following members responded, viz.: Drs. Amling, C. C. Cattanach, J. S. Cattanach, J. S. Cattanach, Jr., Dickson, Delaney, Ellis, Ferster, Foy, Giffen, Gill, Huidekoper, Hanson, Jackson, Lamkin, Lellman, MacKellar, Neher, O'Shea, Ryder, Serling, and Turner (22). The minutes of the last meeting were then read and approved. The minutes of the December meeting were next read and, after slight correction, approved.

Dr. Gill, Chairman of the Board of Censors, reported that the Board recommend that Dr. Johnson be notified to be prepared to meet charges at the next regular meeting. It was then moved and seconded that the Secretary be authorized to formulate charges against S. K. Johnson and forward same to him, and that the Secretary notify Drs. Turner and Finley to have all their evidence ready to be acted upon at the next regular meeting.

The Board of Censors also recommended that a resolution be sent to the Board of Health to the effect that all appointments for milk- or meat-inspectors be made from graduates of veterinary colleges. Moved and seconded that the President appoint a committee of five to attend to it; carried. The Board of Censors also recommended that the Society endeavor to hold its meetings at the Academy of Medicine, suggesting that such a course would add to its prestige. It was then moved and seconded that the President should appoint a committee of three to ascertain the cost of a room in the Academy of Medicine suitable for the Society's meetings, and report at the next meeting; carried.

Dr. O'Shea, Chairman of the Committee on Legislation, then gave a lengthy report of the able work done by that Committee, assuring the members that the Extension Bill for registration was killed, at least for this year, and that the Jury Bill would probably be through by the next meeting. It was then moved and seconded that the report be accepted and placed on file; carried. Moved and seconded that a vote of thanks be extended to the Committee on Legislation; carried.

Dr. Lellman then read a paper on "Calcified Nodes in Lungs of Horses," which was afterward discussed by the members.

The President then appointed the following committees, viz.: As a committee to draft resolutions to the Board of Health in reference to the appointment of meat-inspectors, Drs. H. D. Gill, J. S. Cattanach, H. Neher, J. E.

Ryder, and F. W. Turner and as a committee of three to ascertain the cost of a room at the Academy of Medicine, Drs. H. D. Hanson, J. H. Ferster, and T. Delaney. Moved and seconded that a vote of thanks be extended to Dr. Lellman for his paper; carried. Moved and seconded that the meeting adjourn; carried.

R. W. ELLIS, D.V.S.,
Secretary.

